# Volatile Compounds and Physicochemical Quality of Four Jabuticabas (*Plinia* sp.)

**DOI:** 10.3390/molecules25194543

**Published:** 2020-10-03

**Authors:** Thais Pádua Freitas, Isabela Barroso Taver, Poliana Cristina Spricigo, Lucas Bueno do Amaral, Eduardo Purgatto, Angelo Pedro Jacomino

**Affiliations:** 1 Department of Crop Science, University of São Paulo (USP), Piracicaba 13418-900, Brazil; thaispadua777@gmail.com (T.P.F.); isabela.taver@usp.br (I.B.T.); polianaspricigo@usp.br (P.C.S.); 2 Department of Food Science, University of São Paulo (USP), São Paulo 05508-000, Brazil; lucasbamaral@usp.br (L.B.d.A.); epurgatt@usp.br (E.P.)

**Keywords:** postharvest, aroma, SPME

## Abstract

The jabuticaba is a native Brazilian fruit that has aroused worldwide interest in terms of its nutritional composition and biological activity. However, research on the profile of volatile compounds (VOCs) emitted by these fruits is rare. This study presents the first identification of VOCs from four jabuticaba species. The aim of the study was to characterize the aromatic profile of the following species: ‘Sabará’ (*Plinia jaboticaba*), ‘Escarlate’ (*Plinia phitrantha × Plinia cauliflora*), ‘Otto Andersen’ (*Plinia cauliflora*), and ‘Esalq’ (*Plinia phitrantha*). The analysis was performed by headspace solid-phase microextraction combined with gas chromatography/mass spectrometry (SPME-GC-MS). Multivariate analysis techniques applying the partial least squares-discriminant analysis (PLS-DA) and heatmap were used to compare the results. Fruit quality parameters were determined in terms of fresh mass (g), skin color, soluble solids, and titratable acidity. A total of 117 VOCs was identified including terpenoids, esters, alcohols, aldehydes, alkanes, ketones, and carboxylic acids, with 36 VOCs common to all four species. Terpenes were the majority for all jabuticabas with smaller contributions from other volatile classes, especially β-cubebene, β-elemene, and D-limonene for the ‘Otto Andersen’ jabuticaba.

## 1. Introduction

Jabuticaba (*Plinia* sp.) is a native Brazilian fruit belonging to the botanical family Myrtaceae, naturally occurring in the Atlantic Rainforest biome. This fruit is a globose berry about 2.0 to 3.5 cm in diameter, which develops attached to the stem. When ripe, its skin may display various colors, such as red, dark purple, and black. The pulp has a pleasant taste and is white, gelatinous, sweet exhibiting low acidity, and may contain from one to four seeds [1,2]. Currently, about nine species of jabuticaba are known, as well as some varieties, among them *Plinia jaboticaba*, *Plinia cauliflora*, *Plinia trunciflora*, *Plinia coronate,* and *Plinia phitrantha* [3]. The jabuticaba has aroused the interest of the food industry, due to the presence of high content of compounds exhibiting antioxidant, anti-inflammatory, and anti-hypercholesterolemic activities, mainly anthocyanins and tannins [4,5,6].

‘Sabará’ jabuticabas (*Plinia jaboticaba*) are the most cultivated in Brazil. The fruit is black, with a thin and smooth skin, and a very sweet flavor. The ‘Escarlate’ (*Plinia phitrantha* × *Plinia cauliflora*) jabuticaba presents side fruits [7], with a scarlet-red color, juicy, and sweet pulp. Fruits belonging to the ‘Otto Andersen’ (*Plinia cauliflora*) species are black, with abundant pulps and very sweet. The ‘Esalq’ (*Plinia phitrantha*) jabuticaba is one of the largest of their kind, reaching up to 4 cm in diameter. It is a species developed by the “Luiz de Queiroz” School of Agriculture hence its name, ‘Esalq’. It ranges from reddish to vinaceous, with a sweet and velvety pulp.

Alongside nutritional characteristics and an attractive appearance, the fruit quality perception is determined by fruit volatile organic compound (VOC) profiles [8]. The differential emission of VOCs changes mainly in relation to the interaction of plant organs with their environment, and in the case of fruits, will develop according to the maturation progress [9]. This volatile composition creates an olfactory identity specific to each fruit species and may be applied to distinguish fruits within the same species. Varieties and/or accessions belonging to the same species can share and contain exclusive compounds, which will potentially influence their consumption preference. The VOC profile variability in accessions of the same species has been reported for uvaias, another native Atlantic Rainforest fruit [10].

Volatile monoterpenes such as β-ocymene and linalool, as well as sesquiterpene δ-cadinene, have been described for *Plinia cauliflora* and *Plinia trunciflora* [11]. Terpenes have been described as an abundant class of volatiles in *Plinia jaboticaba* (Vell.) O. Berg, followed by compounds classified as organic acids and alcohols [12]. It is known that the essential jabuticaba oil contains terpenes, such as α-pinene, β-pinene, 1,8-cineole, α-terpineol, and β-caryophyllene, known for their antibacterial and anti-inflammatory actions, suggesting that the same volatile compounds present in the fruit aroma are indicative of endogenous levels and potential bioactive action [13,14,15,16,17].

VOC profile descriptions for jabuticabas are scarce, especially with regard to this fruits’ diversity of species and varieties. In this context, this study is the first to address VOC characterization in ‘Sabará’ (*Plinia jaboticaba*), ‘Escarlate’ (*Plinia phitrantha* × *Plinia cauliflora*), ‘Otto Andersen’ (*Plinia cauliflora*), and ‘Esalq’ (*Plinia phitrantha*) jabuticabas.

## 2. Results and Discussion

The four jabuticaba species were characterized in terms of basic fruit quality attributes (Supplementary material Table S1). The ‘Otto Andersen’ (*P*. *cauliflora*) jabuticaba was noteworthy for presenting a greater fresh mass (11.6 ± 0.3 g), as well as a larger size compared to the other species (height: 25.7 ± 0.7 mm, diameter: 26.6 ± 0.3 mm). The same specie exhibited the highest soluble solids concentration of 19.9 ± 0.2 °Brix. This value corresponds to 1.8-fold the lowest measured value (11.2 ± 0.0 °Brix), in the ‘Sabará’ species. This index can also be compared to Crimson Seedless table grapes (*Vitis vinifera*) [18], considered a fruit with a markedly sweet flavor. The dark purple color of the fruit was characterized by the low hue angle and brightness for all species. The ‘Esalq’ jabuticaba is noteworthy for its lowest color angle (30.3 ± 2.2) and highest brightness (30.3 ± 1.2). The chromaticity, value a* and b* of the fruit skin were up to 3-fold lower in the ‘Otto Andersen’ and ‘Sabará’ jabuticabas in relation to the ‘Scarlet’ and ‘Esalq’ fruits.

**Figure 1:**
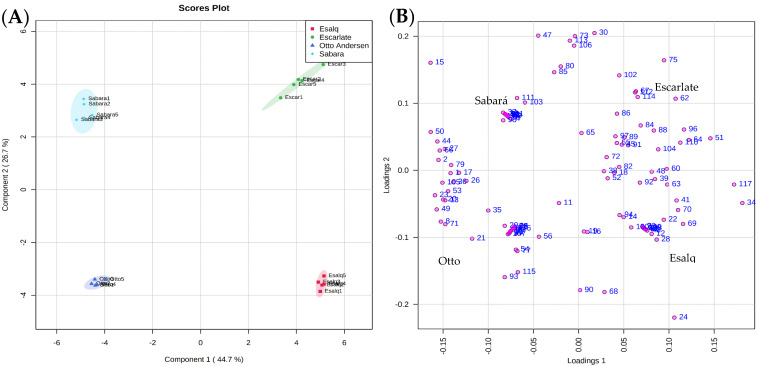

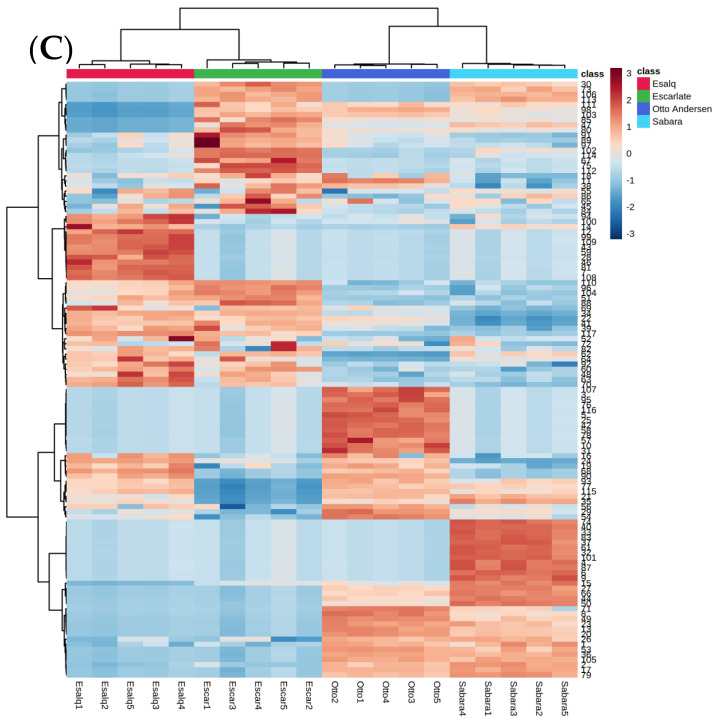
Volatile compound profiles produced by ‘Sabará’ (*Plinia jaboticaba*), ‘Escarlate’ (*Plinia phitrantha × Plinia cauliflora)*, ‘Otto Andersen’ (*Plinia cauliflora*), and ‘Esalq’ (*Plinia phitrantha*) jabuticabas: (**A**) Partial least squares-discriminant analysis (PLS-DA), (**B**) PLSDA loadings, and (**C**) heatmap. The numbers in Figure 1B,C correspond to the code of compounds listed in Table 1. The separation of clusters is shown in detail in Table S2.

The VOC production by the four jabuticaba species totaled 117 distinct compounds (Table 1). The partial least squares-discriminant analysis (PLS-DA) was performed to provide an overview of the aromatic profile of jabuticabas (Figure 1B and Table S2). The main contribution of the PLS-DA was the characterization of the volatile compounds emitted by each jabuticaba, indicating a characteristic aromatic profile for each of the evaluated species, however, the PLS-DA also allowed identifying groups of volatiles shared by the four jabuticabas (Figure 1A).

The principal component analysis displays each species of jabuticaba allocated in a quadrant of the factorial plane, evidencing the dissimilarity between them (Figure 1A,B). PC1 and PC2 components were responsible for 71.4% of the total data variability, being 44.7% (PC1) and 26.7% (PC2) (Figure 1A). Observing Figure 1C, it is possible to group the jabuticabas ‘Otto Andersen’ and ‘Sabará’ as well as ‘Escarlate’ and ‘Esalq’ in two groups by the similarity between the volatile compound’s profiles. Moreover, considering that ‘Escarlate’ jabuticaba is the result of a breeding of the species ‘Otto Andersen’ and ‘Esalq’, the greater influence of ‘Esalq’ on the constitution of the aromatic profile than ‘Otto Andersen’ is evident.

The ‘Sabará’ (*P. jaboticaba*) jabuticaba is the most common and most commercialized species in Brazil. This species presented 12 exclusive VOCs grouped in cluster 1–C1 (Figure 1B): Propyl acetate, cyclofenchene, ethyl isovalerate, ethyl 3-hexenoate, (*Z*)-3-hexen-1-ol acetate, butyl 2-butenoate, cyclopropanecarboxylic acid,3-methylbutyl ester, (*Z*)-3-hexenyl (*E*)-2-butenoate, nonanol, 2-phenylacetamide, n-(1-phenyl-2-propyl), citronellyl butyrate, ethyl cinnamate—most of which are esters, with descriptive notes such as fruity, green, and sweet (Table S2). Esters are widely produced in soft-fleshed fruits during ripening, with the dual function of attracting animals and protecting the fruits against pathogens [19]. Esters are also the most important VOC class in strawberries, for example, where they are responsible for over 90.0% of the ripe fruit’s aroma [20]. Acetate esters, such as (*Z*)-3-hexenyl acetate, are positively evaluated by consumers regarding pear acceptance in sensory analyses, and have been described in ‘Sabará’ jabuticaba [21]. Additionally, acetate esters are involved in defense mechanisms against abiotic stresses in peaches,, which may suggest that they also play this role in jabuticaba [22]. Three ethyl esters were identified exclusively in ‘Sabará’ jabuticaba. This class of compounds has been described as one of the main components of the aroma of the *Passiflora* genus [19]. Aliphatic esters were the major contributors to the aroma of this fruit, indicating that the lipoxygenase (LOX) pathway, which produces sequential lipid degradation, is more active than in the other assessed species. The ‘Escarlate’ jabuticaba (*P. phitrantha × P. cauliflora)* presented the smaller C2 comprising three compounds: α-muurolene, β-patchoulene, 3,6-dimethyl-4h-furo[3,2-c]pyran-4-one (Table S2), the sesquiterpene α-muurolene are the only one with aromatic notes described in the literature. The ‘woody-like’ aroma of this compound is widely emitted by fruits and, mainly, by flowers of different species [23,24,25,26,27]. It has also been identified in fig species, and in *Ficus racemosa*, characterized by being one of the volatiles emitted during the daytime, with the function of attracting pollinating wasps [28,29]. For the ‘Escarlate’ jabuticaba these sesquiterpenes contributed with 4.4% of the total aroma emitted, differentiating it from the other jabuticabas, where α-muurolene was not identified.

The group of volatiles that characterize the ‘Otto Andersen’ (*P. cauliflora*) jabuticaba was categorized in cluster 3. This composition was formed by ethyl propionate, d-α-pinene, ethyl-2-methyl, (*E*)-ethyl tiglate, *cis*-1,3,3-trimethylbicyclo[3.1.0]hexane-1-carboxaldehyde, cyclopentane, 1-ethyl-1-methyl, δ-selinene, l-bornyl acetate, viridiflorene, α-selinene, methyl cinnamate, methyl isoeugenol, β-eudesmol, including monoterpenes, esters, aldehydes, and alkanes, with an emphasis on the most part consisting of sesquiterpenes, sesquiterpenoid esters, and sesquiterpenoid alcohols. Among the 13 VOCs, four have no specific aromatic contribution, three display a pinus-wood-like aroma, and six add a mixture of fruity, floral, and sweet odors. The citrus aroma with a spicy touch of α-selinene sesquiterpene is the most representative component of the cluster, with 1.7% of the total profile emitted by ‘Otto Andersen’ jabuticabas. α -selinene is considered to be one of the characteristic compounds of the aroma of Myrtaceae family species [30,31,32], but has also been identified in other botanical families, such as Cyperaceae and Lamiaceae [33,34].

The VOCs α-thujene, undecane, 3-octanone, (*E*)-2-octenal, 2,2-dimethylhexanal, (*E*)-2-octen-1-ol, benzyl acetate, p-vinylbenzohydrazide, 2,9-bornanediol, and α-eudesmol occur exclusively in ‘Esalq’ jabuticaba (*P. phitrantha*), cluster 4 (Figure 1B). All of them were identified at mean amounts of 0.2% of the total emitted by this species, except for α-thujene (1.4%). Therefore, the most abundant aroma in this cluster was characterized by the pungent ‘green-herbal-woody’ notes attributed to α-thujene. The monoterpene was characterized in cagaita (*Eugenia dysenterica*) [35], eucaliptus (*Eucalyptus* spp.*)* [36], and corresponds to 70.0% of the aroma of ylang-ylang (*Cananga odorata*) and frankincense (*Boswellia* spp.), both traditionally used for therapeutic purposes [37].

**Figure 2:**
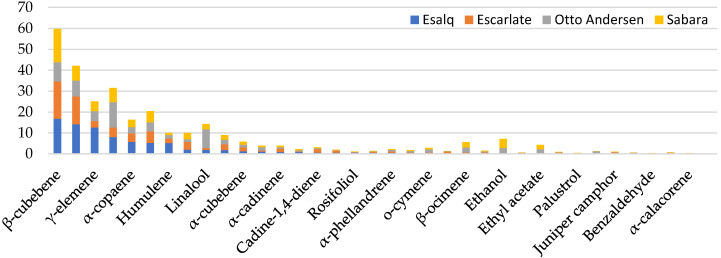
Percentage of the relative area of the 36 volatile organic compounds in relation to all compounds identified in ‘Sabará’ (*Plinia jaboticaba*), ‘Escarlate’ (*Plinia phitrantha × Plinia cauliflora)*, ‘Otto Andersen’ (*Plinia cauliflora*), and ‘Esalq’ (*Plinia phitrantha*) jabuticabas.

The groupings C1, C2, C3, and C4 contain the differential elements that individualize the aromatic profile of the analyzed jabuticaba species (Table S1). These differences could be attributable to the species’ genetic variability, considering that all jabuticabas were grown under the same environmental conditions. On average, the compounds identified represented a small relative area when compared to the total emitted volatiles. The participation of these VOCs in the formation of jabuticaba flavor can contribute to the composition of aroma nuances, while the contribution of the aroma base may be attributed to the most abundant compounds. The compounds with a greater relative area were common to the all four species. In total, 36 VOCs belonging to various classes were identified, namely (14) sesquiterpenes, (9) sesquiterpene alcohols, (5) monoterpenes, (3) alcohols, (2) aldehydes, (1) monoterpenes alcohols, and (1) esters (Figure 2).

Terpenoids were the major volatile organic compounds in the aroma of ‘Sabará’, ‘Escarlate’, ‘Otto Andersen’, and ‘Esalq’ jabuticabas, representing 81.5% of the total, in accordance with previously reported results [5,12,38]. This result suggests that the mevalonic acid or pyruvate and 3-phosphoglycerate routes, necessary for terpene biosynthesis, are the most active pathways in mature jabuticabas in relation to the other metabolic VOC-synthesizing pathways. The differences identified for the 36 VOCs were quantitative, with some compounds being more abundant in one species compared to another. This result was similar to a study carried out with citrus species [39].

The β-cubebene sesquiterpene was the most emitted volatile compound in the analyzed jabuticabas, with the exception of ‘Otto Andersen’, where it appears as the second most emitted VOC. Its contribution to the total area in each species was of 16.8%, 17.8%, and 15.9% for the ‘Esalq’, ‘Escarlate’, and ‘Sabará’ jabuticabas, respectively. β-cubebene has a pleasant aroma translated as fruity and citrus. This compound is commonly found in the essential oils of several species [40,41] with the third most abundant member of the essential oil being obtained from cinnamon leaves (*Cinnamomum osmophloeum*) [42].

The fresh, herbaceous, and waxy aroma of β-elemene ranks second among the most important emissions for jabuticabas, except for ‘Otto Andersen’, where it appears in the fourth place. β -elemene is a sesquiterpene extensively studied due to its biological functions. However, the anticancer activity is by far its most relevant function. β-elemene is capable of causing apoptosis and inhibiting the proliferation of several cancer cells until the most aggressive brain tumor [43]. Currently, *Curcuma wenyujin*, belonging to the Zingiberaceae family, is the plant most applied to the extraction of β-elemene [44]. Our results suggest that jabuticabas, mainly the ‘Esalq’ and ‘Escarlate’ species, are a natural source of this compound, which displays the potential to be used as a raw material for its extraction, as reported for *Nigella damascena* [45].

D-limonene is the most abundant compound in the ‘Otto Andersen’ jabuticaba, representing 12.1% of the total emitted volatile compounds. This VOC is an important component of fragrances, used in cosmetic, pharmaceutical, and food products due to its ‘citric-like’ aroma [46] typical of oranges. This monoterpene has aroused growing interest with regard to the human health, where over sixty scientific articles have been published regarding its biological activity [47]. It exhibits an anti-inflammatory action [48], and is considered promising for the development of drugs to prevent gastric damage [49]. Other studies have reported its action, and its potential use in chemopreventive procedures against different types of cancers [50,51].

The assessed jabuticabas were characterized as displaying woody notes, due to the presence of the sesquiterpenes α-copaene, γ-elemene, humulene, γ-muurolene, δ-cadinene, γ-cadinene, α-cadinene, α-calacorene, T-cadinol, and α-cadinol. The VOCs γ-muurolene (2.2%), δ-cadinene (5.1%), and α-calacorene (0.1%), were detected in similar amounts in all species. However, compounds such as γ-elemene and humulene were 3.0-fold more present in ‘Esalq’ jabuticabas compared to the ‘Otto Andersen’, ‘Sabará’, and ‘Escarlate’ jabuticabas. These terpenes appear frequently in the aromatic profile of other Myrtaceae members, such as jambolão, pitanga, and uvaia [52,53,54].

Linalool was abundant in ‘Otto Andersen’ jabuticabas, representing 9.0% of the total emitted VOC, 4-fold higher than the average of the other three species. The amount of linalool has been related to the regulatory action of methyl jasmonate acid in other species [55,56,57,58,59]. In addition, differences in the emitted amounts may change its specific functions and interactions with linalool itself or its derivatives, due to its multifunctional nature [60]. Linalool is a sesquiterpenoid alcohol with a floral aroma emitted by flowers, fruits [61], and even fungi [62], widely used in the composition of various fragrances. It is also the key compound of the aroma of conventional fruits such as tomatoes (*Solanum licopersum*) [63], citrus [64], and strawberries (*Fragaria × ananassa*) [65].

The compounds ethyl acetate, β-ocimene, o-cymene, and ethanol were abundant in the ‘Otto Andersen’ and ‘Sabará’ jabuticabas. For ethanol, the average of these two species was 17.3-fold higher than the average of the ‘Escarlate’ and ‘Esalq’ jabuticabas.

The volatiles hexanal, α-phellandrene, hexanol, (*Z*)-3-hexen-1-ol, benzaldehyde, 4-terpineol, 1-epi-bicyclosesquiphellandrene, calamenene, α-calacorene, palustrol, ledol, humulane-1,6- dien-3-ol, mansonone, rosifoliol, selina-6-en-4-ol, T-cadinol, α-cadinol, juniper camphor represented less than 1% for all species. Of these VOCs with less area representativeness, hexanal and 4-terpineol were more emitted in the ‘Otto Andersen’ jabuticaba. 4-carvomenthenol is an ingredient used in cosmetics, fine fragrances, and personal care products, in addition to household cleaning products and detergents [66]. Humulane-1,6-dien-3-ol, selina-6-en-4-ol, juniper camphor, and α-cadinol were more emitted in ‘Escarlate’. Certain VOCs, such as ledol and rosifoliol were highlights for ESALQ jabuticabas. Despite the smaller relative areas these VOCs can influence the sensory and flavor characteristics perceived by consumers, if they are present in relatively low odor thresholds (part per billion, ppb).

Some odoriferous compounds have low odor detection thresholds. Among those reported for the four jabuticaba species, benzaldehyde was observed in relatively low amounts (Figure 2) but may have a significant impact on the aroma due to the low detection threshold (0.093 mg Kg^−1^) (Table S3). Other compounds with a low threshold of detection were identified with larger relative areas, such as D-limonene (0.04 mg Kg^−1^) and linalool (0.001 mg Kg^−1^) (Table S3).

Considering the physicochemical quality and aromatic profile of each of the jabuticabas analyzed, there is great potential and diversity to meet distinct consumer demands, and also support genetic improvement programs. The ‘Otto Andersen’ (*P. cauliflora*) jabuticaba stands out for its fresh mass, size, and concentration of soluble solids compared to other species. The typical dark purple color of the skin of the jabuticaba fruit was slightly more intense in the ‘Esalq’ species (*P. phitrantha*) and can be a positive factor in consumer choice. Regarding the composition of the aromatic profile, jabuticaba ‘Sabará’ is interesting for gathering the greatest number of exclusive esters of sweet and fruity notes. The ‘Escarlate’ jabuticaba is distinguished by the intense presence of terpenes, mainly sesquiterpenes.

Jabuticabas are recognized for the presence of bioactive compounds such as ellagitannins and anthocyanins [67], now adding the existence of significant differences in their aromatic profile. The groupings of the volatile compounds of each jabuticaba suggest that changes in the regulatory aspects of the metabolic routes of each variety occur, mainly related to the concentration and availability of substrates, the type of substrate, and enzymatic activity [68]. The volatile profile of jabuticabas is constituted mainly by terpenes, both in the number of detected compounds and in relative area abundance. Among them, β-cubebene, β-elemene, d-limonene, γ-elemene are potentially characteristic candidates of the aroma of members from the *Plinia* genus. Additionally, it is important to note that volatiles abundant in jabuticaba, such as monoterpene d-limonene and sesquiterpene β-elemene, exhibit therapeutic efficiency proven in several studies. Therefore, jabuticabas can be a reference for studies on new possibilities of the uses of different species, prioritizing the extraction of compounds that can benefit human health.

## 3. Material and Methods

### 3.1. Samples

The ripe jabuticabas from four varieties Sabará, Escarlate, Esalq, and Otto Andersen were harvested in November 2019 in Casa Branca, São Paulo, Brazil, latitude 21°46′26″ S, longitude 47°05′11″ W. After collection, the fruits were frozen at −20 °C and transported to the Laboratory of Postharvest of Horticultural Crops in Piracicaba, São Paulo, Brazil (LPV-ESALQ/USP). The fruits (approximately 1 kg of each jabuticaba) were selected according to the appearance, maturation stage, and absence of defects, to ensure homogeneity and uniformity and were frozen at −80 °C until analysis.

### 3.2. Quality Parameters

Fresh mass was determined on an analytical balance and the results expressed in grams (g). Skin color was obtained with a Minolta^®^ CR-300 colorimeter, with two readings taken on opposite sides of each fruit. The results were expressed as color angle (° Hue), luminosity (L *), chromaticity (C *), a * coordinates and b * coordinates.

The height, diameter, and shape of the fruit were measured with a digital caliper with two readings taken at equidistant points for each attribute. The results were expressed in millimeters (mm).

The soluble solid content in the pulp was quantified using an Atago Palette PR-101 digital refractometer [69]. The results were expressed as ° Brix. The pulp’s titratable acidity was determined by the neutralization titration, using 2.0 g of filtered pulp homogenized in 18 mL of distilled water. The samples were titrated with a standard 1 N sodium hydroxide solution (NaOH) to pH 8.1 [70]. The results were expressed as % of citric acid equivalents.

### 3.3. Volatile Compound Determination

#### 3.3.1. Sample Preparation

A total of 3 g of pulp and skin from 150 fruits of each species of jabuticaba were crushed in an analytical mill (IKA A11) with liquid N_2_ and placed in 20 mL glass flasks containing 7 mL of a 30% (*w*/*v*) NaCl solution. The flasks were then sealed with stainless steel lids with silicone septa and stored at −20 °C for GC-MS analysis.

#### 3.3.2. GC-MS Analysis

The volatile compounds were analyzed applying the solid phase microextraction method (SPME), consisting of five replicates each [71]. The vials were thawed in a water bath at 40 °C, under agitation, for 10 min, to accumulate volatile compounds in the headspace. After the equilibrium time, the septum was punctured with a needle, allowing for exposure of the SPME fiber (divinylbenzene, carboxene, and polydimethylsiloxane 50/30 μm, Supelco, Inc., (Sigma-Aldrich, Belfonte, PA, USA)) for 50 min. Once captured, the volatile compounds were desorbed from the fiber by exposure to the heat of the chromatography injector (200 °C) for 5 min.

A Hewlett-Packard (HP) 6890 chromatograph was used for VOC detection, coupled to an HP mass spectrometer model 5973, with a Supelcowax 10 chromatographic column (30 m, 0.25 mm internal diameter, 0.25 μm film thickness). The temperature program used comprised a temperature ramp of 2 °C/min from 40 to 150 °C. The interface temperature between the chromatograph and the selective mass detector was 230 °C and ionization was performed by electron impact (70 eV) with the ion source kept at 150 °C. VOCs were identified by comparison using NIST2011, (Gaithersburg, USA) and confirmed with spectral data available at MassBank North America (MoNA: http://mona.fiehnlab.ucdavis.edu/). The retention index (IR) calculation was performed with a series of *n-alkanes* (C7-C30, Supelco) with the retention indices reported in the literature and with the mass spectrum of authentic external standards. A pool of the following volatile compounds external standards (Sigma-Aldrich, Belfonte, PA, USA) was prepared and injected in GC-MS to validate the identification of compounds by mass spectral comparison: Ethyl acetate; hexanal; 1-hexanol; (*Z*)-3-hexen-1-ol; 2-hexenal; 1-penten-3-ol; 6-methyl-5-hepten-2-one; 1-pentanol; benzaldehyde; β-myrcene; 2-phenylethanol; δ-cadinene; α-cubebene; β-caryophyllene; d-limonene; geraniol; α-phellandrene; isoamyl acetate; ethanol; 4-terpineol; β-caryophyllene oxide; camphor; humulene; γ-terpinene; α-pinene; β-pinene; ethyl butyrate.

### 3.4. Statistical Analyses

An analysis of variance (one-way ANOVA) and the Tukey test (*p* < 0.05) were applied to the physical-chemical characterization data for comparison between species—sample size (N = 5). A partial least squares-discriminant analysis (PLS-DA) and hierarchical clustering and heatmap, prepared using the Metaboanalyst 4.0 program [72] were used to verify the difference between the VOC profiles.

## Figures and Tables

**Table 1:** List of volatile aroma compounds found in ‘Sabará’ (*Plinia jaboticaba*), ‘Escarlate’ (*Plinia phitrantha × Plinia cauliflora)*, ‘Otto Andersen’ (*Plinia cauliflora*), and ‘Esalq’ (*Plinia phitrantha*) jabuticabas by headspace solid-phase microextraction combined with gas chromatography/mass spectrometry (SPME-GC-MS).

Code	Compound	CAS *	Odor Description **	RI calc ^a^	RI lit ^b^	Class	
1	Ethyl acetate	141-78-6	pineapple	909	907	ester	
2	Ethanol	4-17-5	alcohol	929	929	alcohol	
3	Ethyl propionate	105-37-3	fruity, grape, pineapple	977	977	ester	
4	Propyl acetate	109-60-4	solvent, celery	989	980	ester	
5	d-α-pinene	7785-70-8	pine, turpentine	1001	1011	monoterpene	
6	Cyclofenchene	488-97-1	n.d.	1010	n.d.	other	
7	α-thujene	353313	wood, green, herb	1014	1021	monoterpene	
8	Ethyl butyrate	105-54-4	fruity, juicy, pineapple	1045	1047	ester	
9	Ethyl isovalerate	108-64-5	fruity	1054	1060	ester	
10	Ethyl-2-methyl	7452-79-1	sharp, sweet, fruity	1073	1073	ester	
11	Hexanal	66-25-1	grass, tallow, fat	1080	1084	aldehyde	
12	Undecane	1120-21-4	alkane, wax	1109	1100	alkane	
13	β-pinene	127-91-3	pine, resin, turpentine	1117	1116	monoterpene	
14	β-thujene	28634-89-1	n.d.	1125	n.d.	monoterpene	
15	Isoamyl acetate	123-92-2	fresh, banana, sweet	1132	1132	ester	
16	β-myrcene	123-35-3	balsamic, must, spice	1144	1145	monoterpene	
17	Ethyl (*Z*)-crotonate	6776-19-8	n.d.	1156	n.d.	ester	
18	α-Phellandrene	99-83-2	turpentine, mint, spice	1165	1166	monoterpene	
19	d-limonene	5989-27-5	lemon, orange	1174	1178	monoterpene	
20	β-phellandrene	555-10-2	mint, terpentine	1209	1209	monoterpene	
21	1,8 cineole	470-82-6	eucalyptus	1220	1214	monoterpenoid alcohol	
22	2-hexenal	505-57-7	apple, green	1221	1220	aldehyde	
23	Ethyl hexanoate	123-66-0	apple peel, fruity	1224	1223	ester	
24	γ-terpinene	99-85-4	gasoline, turpentine	1237	1238	monoterpene	
25	(*E*)-ethyl tiglate	5837-78-5	sweet, berry, floral	1239	n.d.	ester	
26	β-ocimene	13877-91-3	sweet, herb	1240	1242	monoterpene	
27	Propyl methacrylate	2210-28-8	n.d.	1241	n.d.	ester	
28	3-octanone	106-68-3	fresh, herbal, lavender	1244	1241	ketone	
29	*o*-cymene	527-84-4	n.d.	1260	1260	monoterpene	
30	Hexyl acetate	142-92-7	fruity, apple	1264	1264	ester	
31	*cis*-1,3,3-trimethylbicyclo[3.1.0]hexane-1-carboxaldehyde	1000365-94-2	n.d.	1338	n.d.	aldehyde	
32	Ethyl 3-hexenoate	2396-83-0	sweet, fruity, pineapple	1339	n.d.	ester	
33	(*Z*)-3-hexen-1-ol acetate	3681-71-8	green, banana	1340	1327	ester	
34	4-hexen-1-ol acetate	72237-36-6	n.d.	1343	n.d.	ester	
35	Methyl heptenone	110-93-0	citrus, green, lemongrass	1344	1342	ketone	
36	Ethyl 2-hexenoate	1552-67-6	fruity, green, sweet	1347	1345	ester	
37	Butyl 2-butenoate	7299-91-4	n.d.	1352	n.d.	ester	
38	Hexanol	111-27-3	resin, flower, green	1362	1360	alcohol	
39	(*Z*)-3-hexen-1-ol	928-96-1	grass	1392	1391	alcohol	
40	Cyclopropanecarboxylic acid,3-methylbutyl ester	1000245-65-3	n.d.	1396	n.d.	ester	
41	(*E*)-2-hexen-1-ol	928-95-0	leaf, green, fruity	1398	1401	alcohol	
42	Cyclopentane, 1-ethyl-1-methyl-	16747-50-5	n.d.	1400	n.d.	alkane	
43	(*E*)-2-octenal	2548-87-0	green, nut, fat, leaf	1407	1408	aldehyde	
44	Ethyl octanoate	106-32-1	fruity, fat	1432	1436	ester	
45	α-cubebene	17699-14-8	herb, wax	1462	1463	sesquiterpene	
46	2,2-dimethylhexanal	996-12-3	n.d.	1478	n.d.	aldehyde	
47	δ-elemene	20307-84-0	wood	1480	1468	sesquiterpene	
48	α-copaene	1000360-33-0	wood, spice	1485	1488	sesquiterpene	
49	Ethyl sorbate	110318-09-7	fruity	1487	n.d.	ester	
50	2-ethyl-1-hexanol	104-76-7	rose, green	1493	1487	alcohol	
51	β-bourbonene	5208-59-3	herbal	1495	1495	sesquiterpene	
52	Benzaldehyde	100-52-7	almond, burnt sugar	1498	1502	aldehyde	
53	Grape butyrate	5405-41-4	marshmallow	1522	1524	ester	
54	Linalool	78-70-6	flower, lavender	1533	1537	sesquiterpenoid alcohol	
55	β-cubebene	13744-15-5	citrus, fruity	1555	1546	sesquiterpene	
56	4-terpineol	562-74-3	pepper, woody, earth	1596	1585	monoterpenoid alcohol	
57	δ-selinene	28624-23-9	n.d.	1601	n.d.	sesquiterpene	
58	l-bornyl acetate	5655-61-8	pine	1603	1600	sesquiterpenoid ester	
59	(*E*)-2-octen-1-ol	18409-17-1	mushroom (soap, plastic)	1604	1590	alcohol	
60	β-elemene	515-13-9	herb, wax, fresh	1610	1595	sesquiterpene	
61	(*Z*)-3-hexenyl (*E*)-2-butenoate	65405-80-3	green	1611	1610	ester	
62	Isoledene	1000156-10-8	n.d.	1622	n.d.	sesquiterpene	
63	γ-elemene	490377	green, wood, oil	1637	1636	sesquiterpene	
64	Alloaromadendrene	25246-27-9	wood	1639	1639	sesquiterpene	
65	1-epi-bicyclosesquiphellandrene	54274-73-6	n.d.	1642	n.d.	sesquiterpene	
66	Aristolene	6831-16-9	n.d.	1644	n.d	sesquiterpene	
67	α-muurolene	31983-22-9	wood	1647	n.d.	sesquiterpene	
68	Methyl benzoate	93-58-3	prune, lettuce, herb, sweet	1651	1640	ester	
69	*cis*-muurola-4(14),5-diene	1000365-95-4	n.d.	1661	n.d.	sesquiterpene	
70	Humulene	6753-98-6	wood	1662	1663	sesquiterpene	
71	Ethyl benzoate	93-89-0	camomile, flower, fruity	1665	1658	ester	
72	γ-muurolene	30021-74-0	herb, wood, spice	1675	1681	sesquiterpene	
73	γ-gurjunene	22567-17-5	musty	1678	n.d	sesquiterpene	
74	Nonanol	143-08-8	fat, green	1678	1666	alcohol	
75	β-patchoulene	514-51-2	n.d.	1681	n.d.	sesquiterpene	
76	Viridiflorene	21747-46-6	n.d.	1699	n.d.	sesquiterpene	
77	β-selinene	17066-67-0	herb	1715	1711	sesquiterpene	
78	α-selinene	473-13-2	pepper, orange	1719	1724	sesquiterpene	
79	(*E*)-germacrene D	23986-74-5	wood, spice	1731	1724	sesquiterpene	
80	α-amorphene	483-75-0	n.d.	1742	1752	sesquiterpene	
81	Benzyl acetate	140-11-4	floral, fruity, jasmin	1755	1747	ester	
82	δ-cadinene	483-76-1	thyme, medicine, wood	1759	1749	sesquiterpene	
83	2-phenylacetamide, *N*-(1-phenyl-2-propyl)-	1000223-70-1	n.d.	1762	n.d.	other	
84	Cadine-1,4-diene	16728-99-7	spice, fruity	1785	1786	sesquiterpene	
85	Selina-3,7(11)-diene	6813-21-4	n.d.	1789	1789	sesquiterpene	
86	γ-cadinene	39029-41-9	wood	1790	1776	sesquiterpene	
87	Citronellyl butyrate	141-16-2	fruity, sweet, rose	1802	1809	monoterpenoid ester	
88	α-cadinene	24406-05-1	woody, dry	1817	1815	sesquiterpene	
89	Calamenene	483-77-2	herb, spice	1824	1822	sesquiterpene	
90	Geraniol	106-24-1	rose, geranium	1845	1847	monoterpenoid alcohol	
91	α-calacorene	21391-99-1	wood	1890	1901	sesquiterpene	
92	Palustrol	95975-84-1	n.d.	1910	n.d.	sesquiterpenoid alcohol	
93	β-caryophyllene oxide	1139-30-6	herb, sweet, spice	2020	2014	sesquiterpene	
94	Ledol	577-27-5	sweet, green	2040	2043	sesquiterpenoid alcohol	
95	Methyl cinnamate	103-26-4	strawberry, cherry	2065	2056	ester	
96	Humulane-1,6-dien-3-ol	1000140-23-1	n.d.	2081	n.d.	sesquiterpenoid alcohol	
97	Mansonone	5574-34-5	n.d.	2095	n.d.	other	
98	Cubenol	21284-22-0	spice, herb, green tea	2097	2097	sesquiterpenoid alcohol	
99	p-vinylbenzohydrazide	1000244-74-9	n.d.	2105	n.d.	other	
100	Rosifoliol	63891-61-2	n.d.	2115	n.d.	sesquiterpenoid alcohol	
101	Ethyl cinnamate	103-36-6	sweet, fruity, spicy, berry plum	2136	2139	ester	
102	Selina-6-en-4-ol	1000140-23-2	n.d.	2141	n.d.	sesquiterpenoid alcohol	
103	Carotol	465-28-1	pleasent mild	2149	n.d.	sesquiterpenoid alcohol	
104	T-cadinol	1474790	wood, balsamic	2150	2155	sesquiterpenoid alcohol	
105	T-muurolol	19912-62-0	herb, weak spice, honey	2157	2148	sesquiterpenoid alcohol	
106	Spathulenol	6750-60-3	earthy, herbal, fruity	2170	2153	sesquiterpenoid alcohol	
107	Methyl isoeugenol	93-16-3	spicy, clove, blossom	2180	2196	other	
108	2,9-bornanediol	54831-21-9	n.d.	2185	n.d.	monoterpenoid alcohol	
109	α-eudesmol	473-16-5	sweet, wood	2190	2208	sesquiterpenoid alcohol	
110	α-cadinol	481-34-5	herb, wood	2192	2191	sesquiterpenoid alcohol	
111	Cadalene	483-78-3	n.d.	2196	2203	sesquiterpene	
112	3,6-Dimethyl-4H-furo[3,2-c]pyran-4-one	36745-38-7	n.d.	2201	n.d.	ketone	
113	Occidentalol	29484-47-7	n.d.	2204	n.d.	sesquiterpenoid alcohol	
114	Juniper camphor	473-04-1	camphor	2207	n.d.	sesquiterpenoid alcohol	
115	Tetracyclo[6.3.2.0(2,5).0(1,8)]tridecan-9-ol, 4,4-dimethyl	1000157-75-1	n.d.	2211	n.d.	alcohol	
116	β-eudesmol	473-15-4	wood, green	2225	2214	sesquiterpenoid alcohol	
117	Galaxolide 2	1000285-26-7	musk	2230	n.d.	other	

* Chemical abstracts service (CAS) is a division of the American Chemical Society. ** Descriptions consulted on the site https://www.pherobase.com/, http://www.thegoodscentscompany.com/, https://www.flavornet.org/, https://cosylab.iiitd.edu.in/flavordb/. ^a^ Retention index calculated. ^b^ Retention index from the literature database https://webbook.nist.gov/chemistry/, https://mona.fiehnlab.ucdavis.edu/, https://www.pherobase.com/.

## Supplementary Materials

The following are available online, Table S1: Physicochemical characterization of jabuticabas Sabará (Plinia jaboticaba), Escarlate (Plinia phitrantha × Plinia cauliflora), Otto Andersen (Plinia cauliflora) e Esalq (Plinia phitrantha); Table S2: Volatile aromatic compounds divided by clusters of each jabuticaba obtained by headspace solid phase microextraction combined with gas chromatography/mass spectrometry (SPME-GC-MS). C1, C2, C3, C4 indicate clusters of compounds exclusive for each species. Where: Sabara: ‘Sabará’ (Plinia jaboticaba), ESCAR: ‘Escarlate’ (Plinia phitrantha × Plinia cauliflora), OTTO: ‘Otto Andersen’ (Plinia cauliflora) and ‘Esalq’ (Plinia phitrantha)
