# Supplementary material for: Volatile Compounds and Physicochemical Quality of Four Jabuticabas (Plinia sp.)
Source: Molecules. 2020 Oct 3;25(19):4543. doi: 10.3390/molecules25194543 (PMC7582703; doi:10.3390/molecules25194543)
Supplement: Supplementary file 1 [file molecules-25-04543-s001.pdf]

Supplementary Materials:

Table S1: Physicochemical characterization of jabuticabas Sabará (*Plinia jaboticaba*), Escarlate (*Plinia phitrantha x Plinia cauliflora*), Otto Andersen (*Plinia cauliflora*) e Esalq (*Plinia phitrantha*).

**Jabuticaba**	**Mass**	**Height**	**Diameter**	**Soluble Solids**	**Titratble acidity**	**Skin color**				
	**(g)**	**(mm)**	**(mm)**	**(° *Brix*)**	**(% citric acid)**	**°*Hue***	**L***	**C***	***a****	***b****
Sabará (*Plinia jaboticaba*)	7,37 ± 0,25 [a]	23,37 ± 0,47 [a]	23,30 ± 0,19 [a]	11,23 ± 0,05 [a]	0,52 ± 0,02 [a]	33,99 ± 2,40 [a, b]	25,64 ± 0,08 [a]	7,16 ± 1,77 [a]	6,97 ± 0,59 [a]	4,76 ± 0,74 [a]
Escarlate (*Plinia phitrantha x Plinia cauliflora*)	7,01 ± 0,30 [a]	21,95 ± 0,72 [a]	22,40 ± 0,54 [a]	14,58 ± 0,22 [c]	1,83 ± ,028 [b]	35,90 ± 1,25 [b]	28,88 ± 0,89 [b,c]	19,08 ± 0,38 [b]	17,96 ± 0,27 [b]	12,98 ± 0,38 [c]
Otto Andersen (*Plinia cauliflora*)	11,60 ± 0,33 [b]	25,72 ± 0,77 [b]	26,62 ± 0,31 [b]	19,98 ± 0,25 [d]	1,60 ± 0,10 [b]	35,64 ± 0,39 [a,b]	27,28 ± 0,54 [a,b]	6,65 ± 1,58 [a]	6,33 ± 0,36 [a]	4,55 ± 0,30 [a]
Esalq (*Plinia phitrantha*)	7,38 ± 0,19 [a]	22,57 ± 0,52 [a]	22,71 ± 0,90 [a]	12,16 ± 0,12 [b]	2,00 ± 0,13 [b]	30,37 ± 2,24 [a]	30,31 ± 1,29 [c]	17,17 ± 6,23 [b]	18,02 ± 1,17 [b]	10,55 ± 0,57 [b]

Means followed by the same letter do not differ from each other by one-way ANOVA and Tukey’s test (n=15) ($p<0.05$).

Table S2: Volatile aromatic compounds divided by clusters of each jabuticaba obtained by headspace solid phase microextraction combined with gas chromatography/mass spectrometry (SPME-GC-MS). C1, C2, C3, C4 indicate clusters of compounds exclusive for each species. Where: Sabara: 'Sabará' (*Plinia jaboticaba),* ESCAR: 'Escarlate' (*Plinia phitrantha* × *Plinia cauliflora*), OTTO: 'Otto Andersen' (*Plinia cauliflora*) and 'Esalq' (*Plinia phitrantha*)

Code	Compound	CAS	Odor description	RI calc	RI lit	Class	Clusters
4	Propyl acetate	109-60-4	solvent, celery	989	980	ester	C1-SABARA
6	Cyclofenchene	488-97-1	n.d.	1010	n.d.	other	C1-SABARA
9	Ethyl isovalerate	108-64-5	fruity	1054	1060	ester	C1-SABARA
33	Ethyl 3-hexenoate	2396-83-0	sweet, fruity, pineapple	1339	n.d.	ester	C1-SABARA
34	(Z)-3-hexen-1-ol acetate	3681-71-8	green, banana	1340	1327	ester	C1-SABARA
38	Butyl 2-butenoate	7299-91-4	n.d.	1352	n.d.	ester	C1-SABARA
41	Cyclopropanecarboxylic acid,3-methylbutyl ester	1000245-65-3	n.d.	1396	n.d.	carboxilic acid	C1-SABARA
62	(Z)-3-hexenyl (E)-2-butenoate	65405-80-3	green	1611	1610	ester	C1-SABARA
77	Nonanol	143-08-8	fat, green	1678	1666	alcohol	C1-SABARA
86	2-phenylacetamide, N-(1-phenyl-2-propyl)-	1000223-70-1	n.d.	1762	n.d.	other	C1-SABARA
90	Citronellyl butyrate	141-16-2	fruity, sweet, rose	1802	1809	monoterpenoid ester	C1-SABARA
106	Ethyl cinnamate	103-36-6	balsam, fruity, spicy, berry, plum	2136	2139	ester	C1-SABARA
70	α-Muurolene	31983-22-9	wood	1647	n.d.	sesquiterpene	C2-ESCAR
78	β-patchoulene	514-51-2	n.d.	1681	n.d.	sesquiterpene	C2-ESCAR
117	3,6-dimethyl-4H-furo[3,2-c]pyran-4-one	36745-38-7	n.d.	2201	n.d.	ketone	C2-ESCAR
3	Ethyl propionate	105-37-3	fruity, grape, pineapple	977	977	ester	C3-OTTO
5	D-α-pinene	7785-70-8	pine, turpentine	1001	1011	monoterpene	C3-OTTO
10	Ethyl-2-methyl	7452-79-1	sharp, sweet, fruity	1073	1073	ester	C3-OTTO
25	(E)-ethyl tiglate	5837-78-5	sweet, tropical, berry, floral	1239	n.d.	ester	C3-OTTO
32	cis-1,3,3-trimethylbicyclo[3.1.0]hexane-1-carboxaldehyde	1000365-94-2	n.d.	1338	n.d.	aldehyde	C3-OTTO
43	Cyclopentane, 1-ethyl-1-methyl-	16747-50-5	n.d.	1400	n.d.	alkane	C3-OTTO

58	δ-selinene	28624-23-9	n.d.	1601	n.d.	sesquiterpene	C3-OTTO
59	L-bornyl acetate	5655-61-8	pine	1603	1600	sesquiterpenoid ester	C3-OTTO
79	Viridiflorene	21747-46-6	n.d.	1699	n.d.	sesquiterpene	C3-OTTO
81	α-selinene	473-13-2	pepper, orange	1719	1724	sesquiterpene	C3-OTTO
98	Methyl cinnamate	103-26-4	strawberry, cherry, cinnamon	2065	2056	ester	C3-OTTO
112	Methyl isoeugenol	93-16-3	spicy, clove, blossom, carnation	2180	2196	other	C3-OTTO
121	β-eudesmol	473-15-4	wood, green	2225	2214	sesquiterpenoid alcohol	C3-OTTO
7	α-thujene	2867-05-2	wood, green, herb	1014	1021	monoterpene	C4-ESALQ
12	Undecane	1120-21-4	alkane, wax	1109	1100	alkane	C4-ESALQ
28	3-octanone	106-68-3	fresh, herbal, lavender	1244	1241	ketone	C4-ESALQ
44	(E)-2-octenal	2548-87-0	green, nut, fat, leaf, walnut	1407	1408	aldehyde	C4-ESALQ
47	2,2-dimethylhexanal	996-12-3	n.d.	1478	n.d.	aldehyde	C4-ESALQ
60	(E)-2-octen-1-ol	18409-17-1	mushroom (soap, plastic)	1604	1590	alcohol	C4-ESALQ
84	Benzyl acetate	140-11-4	floral, fruity, jasmin	1755	1747	ester	C4-ESALQ
103	p-vinylbenzohydrazide	1000244-74-9	n.d.	2105	n.d.	other	C4-ESALQ
113	2,9-bornanediol	54831-21-9	n.d.	2185	n.d.	monoterpenoid alcohol	C4-ESALQ
114	α-eudesmol	473-16-5	sweet, wood	2190	2208	sesquiterpenoid alcohol	C4-ESALQ

* Chemical Abstracts Service (CAS) is a division of the American Chemical Society. ** Descriptions consulted on the site http://www.thegoodscentscompany.com/.

[a] Retention index calculated. [B] Retention index from literature from database https://mona.fiehnlab.ucdavis.edu/.

Table S3: Odor thresholds of **v**olatiles compounds of jabuticabas Sabará (*Plinia jaboticaba*), Escarlate (*Plinia phitrantha x Plinia cauliflora*), Otto Andersen (*Plinia cauliflora*) e Esalq (*Plinia phitrantha*).

Code	Compound	CAS	Odor description	Class	Odor treshold (mg. Kg^{-1})
1	Ethyl acetate	141-78-6	pineapple	ester	1.190[1]
2	Ethanol	4-17-5	alcohol	alcohol	0.090[2]
3	Ethyl propionate	105-37-3	fruity, grape, pineapple	ester	3.452[3]
4	Propyl acetate	109-60-4	solvent, celery	ester	0.239[4]
5	D-α-pinene	7785-70-8	pine, turpentine	monoterpene	0.190[5]
6	Cyclofenchene	488-97-1	n.d.	other	-
7	α-thujene	353313	wood, green, herb	monoterpene	22.000[6]
8	Ethyl butyrate	105-54-4	fruity, juicy, pineapple	ester	0.400[7]
9	Ethyl isovalerate	108-64-5	fruity	ester	0.001[3]
10	Ethyl-2-methyl	7452-79-1	sharp, sweet, fruity	ester	0.968-11.700[8]
11	Hexanal	66-25-1	grass, tallow, fat	aldehyde	75.000[5]
12	Undecane	1120-21-4	alkane, wax	alkane	5.600[4]
13	β-pinene	127-91-3	pine, resin, turpentine	monoterpene	1.500[5]
14	β-thujene	28634-89-1	n.d.	monoterpene	-
15	Isoamyl acetate	123-92-2	fresh, banana, sweet	ester	0.013 - 0.172[9]
16	β-myrcene	123-35-3	balsamic, must, spice	monoterpene	0.100[5]
17	Ethyl (Z)-crotonate	6776-19-8	n.d.	ester	-
18	α-Phellandrene	99-83-2	turpentine, mint, spice	monoterpene	0.160[10]
19	D-limonene	5989-27-5	lemon, orange	monoterpene	0.040[4]
20	β-phellandrene	555-10-2	mint, terpentine	monoterpene	0.500[11]
21	1,8 cineole	470-82-6	eucalyptus	monoterpenoid alcohol	0.001[12]
22	2-hexenal	505-57-7	apple, green	aldehyde	424.000[13]
23	Ethyl hexanoate	123-66-0	apple peel, fruity	ester	0.080[7]
24	γ-Terpinene	99-85-4	gasoline, turpentine	monoterpene	0.260[5]
25	(E)-Ethyl tiglate	5837-78-5	sweet, berry, floral	ester	0.063[14]

26	β-ocimene	13877-91-3	sweet, herb	monoterpene	0.034[5]
27	Propyl methacrylate	2210-28-8	n.d.	ester	-
28	3-octanone	106-68-3	fresh, herbal, lavender	ketone	0.057[15]
29	o-cymene	527-84-4	n.d.	monoterpene	0.004[16]
30	Hexyl acetate	142-92-7	fruity, apple	ester	0.237[17]
31	cis-1,3,3-trimethylbicyclo[3.1.0]hexane-1-carboxaldehyde	1000365-94-2	n.d.	aldehyde	-
32	Ethyl 3-hexenoate	2396-83-0	sweet, fruity, pineapple	ester	-
33	(Z)-3-hexen-1-ol acetate	3681-71-8	green, banana	ester	0.013[12]
34	4-hexen-1-ol acetate	72237-36-6	n.d.	ester	-
35	Methyl heptenone	110-93-0	citrus, green, lemongrass	ketone	0.019[18]
36	Ethyl 2-hexenoate	1552-67-6	fruity, green, sweet	ester	-
37	Butyl 2-butenoate	7299-91-4	n.d.	ester	-
38	Hexanol	111-27-3	resin, flower, green	alcohol	0.180[19]
39	(Z)-3-hexen-1-ol	928-96-1	grass	alcohol	0.910[5]
40	Cyclopropanecarboxylic acid,3-methylbutyl ester	1000245-65-3	n.d.	ester	-
41	(E)-2-hexen-1-ol	928-95-0	leaf, green, fruity	alcohol	0.100[5]
42	Cyclopentane, 1-ethyl-1-methyl-	16747-50-5	n.d.	alkane	-
43	(E)-2-octenal	2548-87-0	green, nut, fat, leaf	aldehyde	0.003[5]
44	Ethyl octanoate	106-32-1	fruity, fat	ester	0.580[7]
45	α-cubebene	17699-14-8	herb, wax	sesquiterpene	-
46	2,2-dimethylhexanal	996-12-3	n.d.	aldehyde	-
47	δ-elemene	20307-84-0	wood	sesquiterpene	-
48	α-copaene	1000360-33-0	wood, spice	sesquiterpene	-
49	Ethyl sorbate	110318-09-7	fruity	ester	-
50	2-Ethyl-1-hexanol	104-76-7	rose, green	alcohol	0.400[17]
51	β-bourbonene	5208-59-3	herbal	sesquiterpene	-
52	Benzaldehyde	100-52-7	almond, burnt sugar	aldehyde	0.093[20]
53	Grape butyrate	5405-41-4	marshmallow	ester	2.500[21]
54	Linalool	78-70-6	flower, lavender	sesquiterpenoid alcohol	0.001[5]
55	β-cubebene	13744-15-5	citrus, fruity	sesquiterpene	-
56	4-terpineol	562-74-3	pepper, woody, earth	monoterpenoid alcohol	32.000[5]
57	δ-selinene	28624-23-9	n.d.	sesquiterpene	-

58	L-bornyl acetate	5655-61-8	pine	sesquiterpenoid ester	-
59	(E)-2-octen-1-ol	18409-17-1	mushroom (soap, plastic)	alcohol	0.840[22]
60	β-elemene	515-13-9	herb, wax, fresh	sesquiterpene	-
61	(Z)-3-hexenyl (E)-2-butenoate	65405-80-3	green	ester	0.500[5]
62	Isoledene	1000156-10-8	n.d.	sesquiterpene	-
63	γ-elemene	490377	green, wood, oil	sesquiterpene	-
64	Alloaromadendrene	25246-27-9	wood	sesquiterpene	-
65	1-epi-bicyclosesquiphellandrene	54274-73-6	n.d.	sesquiterpene	-
66	Aristolene	6831-16-9	n.d.	sesquiterpene	-
67	α-muurolene	31983-22-9	wood	sesquiterpene	-
68	Methyl benzoate	93-58-3	prune, lettuce, herb, sweet	ester	0.073[23]
69	cis-muurola-4(14),5-diene	1000365-95-4	n.d.	sesquiterpene	-
70	Humulene	6753-98-6	wood	sesquiterpene	0.390[5]
71	Ethyl benzoate	93-89-0	camomile, flower, fruity	ester	0.620[16]
72	γ-muurolene	30021-74-0	herb, wood, spice	sesquiterpene	-
73	γ-gurjunene	22567-17-5	musty	sesquiterpene	-
74	Nonanol	143-08-8	fat, green	alcohol	0.045[24]
75	β-patchoulene	514-51-2	n.d.	sesquiterpene	-
76	Viridiflorene	21747-46-6	n.d.	sesquiterpene	-
77	β-selinene	17066-67-0	herb	sesquiterpene	-
78	α-selinene	473-13-2	pepper, orange	sesquiterpene	-
79	(E)-germacrene D	23986-74-5	wood, spice	sesquiterpene	-
80	α-amorphene	483-75-0	n.d.	sesquiterpene	-
81	Benzyl acetate	140-11-4	floral, fruity, jasmin	ester	14.000 - 22.000[25]
82	δ-cadinene	483-76-1	thyme, medicine, wood	sesquiterpene	-
83	2-phenylacetamide, N-(1-phenyl-2-propyl)-	1000223-70-1	n.d.	other	-
84	Cadine-1,4-diene	16728-99-7	spice, fruity	sesquiterpene	-
85	Selina-3,7(11)-diene	6813-21-4	n.d.	sesquiterpene	-
86	γ-cadinene	39029-41-9	wood	sesquiterpene	-
87	Citronellyl butyrate	141-16-2	fruity, sweet, rose	monoterpenoid ester	-
88	α-cadinene	24406-05-1	woody, dry	sesquiterpene	-
89	Calamenene	483-77-2	herb, spice	sesquiterpene	-

90	Geraniol	106-24-1	rose, geranium	monoterpenoid alcohol	0.001[12]
91	α-calacorene	21391-99-1	wood	sesquiterpene	-
92	Palustrol	95975-84-1	n.d.	sesquiterpenoid alcohol	-
93	β-caryophyllene oxide	1139-30-6	herb, sweet, spice	sesquiterpene	0.41[5]
94	Ledol	577-27-5	sweet, green	sesquiterpenoid alcohol	-
95	Methyl cinnamate	103-26-4	strawberry, cherry	ester	0.12[26]
96	Humulane-1,6-dien-3-ol	1000140-23-1	n.d.	sesquiterpenoid alcohol	-
97	Mansonone	5574-34-5	n.d.	other	-
98	Cubenol	21284-22-0	spice, herb, green tea	sesquiterpenoid alcohol	-
99	p-vinylbenzohydrazide	1000244-74-9	n.d.	other	-
100	Rosifoliol	63891-61-2	n.d.	sesquiterpenoid alcohol	-
101	Ethyl cinnamate	103-36-6	sweet, fruity, spicy, berry plum	ester	0.017-0.04[16]
102	Selina-6-en-4-ol	1000140-23-2	n.d.	sesquiterpenoid alcohol	-
103	Carotol	465-28-1	pleasent mild	sesquiterpenoid alcohol	0.008[27]
104	T-cadinol	1474790	wood, balsamic	sesquiterpenoid alcohol	-
105	T-muurolol	19912-62-0	herb, weak spice, honey	sesquiterpenoid alcohol	-
106	Spathulenol	6750-60-3	earthy, herbal, fruity	sesquiterpenoid alcohol	-
107	Methyl isoeugenol	93-16-3	spicy, clove, blossom	other	1.600[28]
108	2,9-bornanediol	54831-21-9	n.d.	monoterpenoid alcohol	-
109	α-eudesmol	473-16-5	sweet, wood	sesquiterpenoid alcohol	-
110	α-cadinol	481-34-5	herb, wood	sesquiterpenoid alcohol	-
111	Cadalene	483-78-3	n.d.	sesquiterpene	-
112	3,6-Dimethyl-4H-furo[3,2-c]pyran-4-one	36745-38-7	n.d.	ketone	-
113	Occidentalol	29484-47-7	n.d.	sesquiterpenoid alcohol	-
114	Juniper camphor	473-04-1	camphor	sesquiterpenoid alcohol	0.520[10]
115	Tetracyclo[6.3.2.0(2,5).0(1,8)]tridecan-9-ol, 4,4-dimethyl	1000157-75-1	n.d.	alcohol	-
116	β-eudesmol	473-15-4	wood, green	sesquiterpenoid alcohol	-
117	Galaxolide 2	1000285-26-7	musk	other	-

References

1. Ueno, H.; Amano, S.; Merecka, B.; Kośmider, J. Difference in the odor concentrations measured by the triangle odor bag method and dynamic olfactometry. *Water Sci. Technol.* **2009**, *59*, 1339–1342, doi:10.2166/wst.2009.112.
2. Cain, W.S.; de Wijk*, R.A.; Jalowayski, A.A.; Pilla Caminha, G.; Schmidt, R. Odor and chemesthesis from brief exposures to TXIB. *Indoor Air* **2005**, *15*, 445–457.
3. Poisson, L.; Schieberle, P. Characterization of the most odor-active compounds in an American Bourbon whisky by application of the aroma extract dilution analysis. *J. Agric. Food Chem.* **2008**, *56*, 5813–5819, doi:10.1021/jf800382m.
4. Nagata, Y. Measurement of odor threshold by triangle odor bag method. *chemestry* **2003**, 118–127.
5. Tamura, H.; Boonbumrung, S.; Yoshizawa, T.; Varanyanond, W. The volatile constituents in the Peel and Pulp of a green thai mango, Khieo Sawoei cultivar (Mangifera indica L.). *Food Sci. Technol. Res* **2001**, *7*, 72–77, doi:10.3136/fstr.7.72.
6. Pino, J.A.; Quijano, C.E. Estudo de compostos voláteis de ameixa (prunus domestica L. cv. horvin) e estimativa da sua contribuição ao aroma. *Cienc. e Tecnol. Aliment.* **2012**, *32*, 76–83, doi:10.1590/S0101-20612012005000006.
7. Peinado, R.A.; Moreno, J.; Bueno, J.E.; Moreno, J.A.; Mauricio, J.C. Comparative study of aromatic compounds in two young white wines subjected to pre-fermentative cryomaceration. *Food Chem.* **2004**, *84*, 585–590, doi:10.1016/S0308-8146(03)00282-6.
8. Komthong, P.; Katoh, T.; Igura, N.; Shimoda, M. Changes in the odours of apple juice during enzymatic browning. *Food Qual. Prefer.* **2006**, *17*, 497–504, doi:10.1016/j.foodqual.2005.06.003.
9. Atanasova, B.; Thomas-Danguin, T.; Langlois, D.; Nicklaus, S.; Chabanet, C.; Etiévant, P. Perception of wine fruity and woody notes: influence of peri-threshold odorants. *Food Qual. Prefer.* **2005**, *16*, 504–510, doi:10.1016/j.foodqual.2004.10.004.
10. Padrayuttawat, A..; Yoshizawa, T..; Tamura, H..; Tokunaga, T. Optical isomers and odor thresholds of volatile constituents in Citrus sudachi. *Food Sci. Technol. Int. Tokyo* **1997**, *3*, 402–408, doi:10.3136/fsti9596t9798.3.402.
11. Teranishi, R.; Buttery, R.G.; Matsumoto, K.E.; Stern, D.J.; Cunningham, R.T.; Gothilf, S. Recent Developments in Chemical Attractants for Tephritid Fruit Flies. In; 1987; pp. 431–438.
12. Czerny, M.; Christlbauer, M.; Christlbauer, M.; Fischer, A.; Granvogl, M.; Hammer, M.; Hartl, C.; Hernandez, N.M.; Schieberle, P. Re-investigation on odour thresholds of key food aroma compounds and development of an aroma language based on odour qualities of defined aqueous odorant solutions. *Eur. Food Res. Technol.* **2008**, *228*, 265–273, doi:10.1007/s00217-008-0931-x.
13. Kalua, C.M.; Allen, M.S.; Bedgood, D.R.; Bishop, A.G.; Prenzler, P.D.; Robards, K. Olive oil volatile compounds, flavour development and quality: A critical review. *Food Chem.* **2007**, *100*, 273–286, doi:10.1016/j.foodchem.2005.09.059.
14. Takeoka, G.R.; Buttery, R.G.; Ling, L.C.; Wong, R.Y.; Dao, L.T.; Edwards, R.H.; De J Berrios, J. Odor thresholds of various unsaturated branched esters. *LWT - Food Sci. Technol.* **1998**, *31*, 443–448, doi:10.1006/fstl.1998.0382.
15. Siegmund, B.; Pöllinger-Zierler, B. Odor thresholds of microbially induced off-flavor compounds in apple juice. *J. Agric. Food Chem.* **2006**, *54*, 5984–5989, doi:10.1021/jf060602n.
16. van Gemer, L.J. *ODOUR THRESHOLDS*; van Gemert, L.J., Ed.; 2nd ed.; Oliemans Punter & Partners BV: Netherlands, 2011; Vol. 1; ISBN 9789081089401.
17. Hellman, T.M.; Small, F.H. Characterization of the odor properties of 101 petrochemicals using sensory methods. *J. Air Pollut. Control Assoc.* **1974**, *24*, 979–982, doi:10.1080/00022470.1974.10470005.
18. Pickett, J.A.; Birkett, M.A.; Bruce, T.J.A.; Chamberlain, K.; Gordon-Weeks, R.; Matthes, M.C.; Napier, J.A.; Smart, L.E.; Woodcock, C.M. Developments in aspects of ecological phytochemistry: The role of

cis-jasmone in inducible defence systems in plants. *Phytochemistry* **2007**, *68*, 2937–2945, doi:10.1016/j.phytochem.2007.09.025.

19. Ferreira, V.; Ardanuy, M.; López, R.; Cacho, J.F. Relationship between Flavor Dilution Values and Odor Unit Values in Hydroalcoholic Solutions: Role of Volatility and a Practical Rule for Its Estimation. *J. Agric. Food Chem.* **1998**, *46*, 4341–4346, doi:10.1021/jf980144l.
20. Von Ranson, C.; Belitz, H.-D. *Originalarbeit Untersuchungen zur Struktur-Aktivitiitsbeziehung bei Geruchsstoffen 3. Mitteilung: Wahrnehmungs-und Erkennungsschwellenwerte sowie Geruchsqualitiiten alicyclischer und aromatischer Aldehyde* *; 1992; Vol. 195;.
21. Takeoka, G.R.; Buttery, R.G.; Turnbaugh, J.G.; Benson, M. Odor thresholds of various branched esters. *LWT - Food Sci. Technol.* **1995**, *28*, 153–156, doi:10.1016/S0023-6438(95)80028-X.
22. Eriksson, C.E.; Lundgren, B.; Vallentin, K. Odor detectability of aldehydes and alcohols originating from lipid oxidation. *Chem. Senses* **1976**, *2*, 3–15, doi:10.1093/chemse/2.1.3.
23. Steinhaus, M.; Sinuco, D.; Polster, J.; Osorio, C.; Schieberle, P. Characterization of the key aroma compounds in pink guava (Psidium guajawa L.) by means of aroma Re-engineering experiments and Omission Tests. *J. Agric. Food Chem.* **2009**, *57*, 2882–2888, doi:10.1021/jf803728n.
24. Giri, A.; Osako, K.; Okamoto, A.; Ohshima, T. Olfactometric characterization of aroma active compounds in fermented fish paste in comparison with fish sauce, fermented soy paste and sauce products. *Food Res. Int.* **2010**, *43*, 1027–1040, doi:10.1016/j.foodres.2010.01.012.
25. Köster, E.P. Adaptation and cross-adaptation in olfaction: an experimental study with olfactory stimuli at low levels of intensity, Utrecht University: Utrecht, 1971.
26. Ismail, H.M.; Williams, A.A.; Tucknott, O.G. The flavour of plums (Prunus domestica L.). An examination of the aroma components of plum juice from the cultivar victoria. *J. Sci. Food Agric.* **1981**, *32*, 613–619, doi:10.1002/jsfa.2740320614.
27. Buttery, R.G.; Seifert, R.M.; Guadagni, D.G.; Black, D.R.; Ling, L.C. Characterization of some Volatile constituents of carrots. *J. Agric. Food Chem.* **1968**, *16*, 1009–1015, doi:10.1021/jf60160a012.
28. Zeller, A.; Rychlik, M. Character impact odorants of fennel fruits and fennel tea. *J. Agric. Food Chem.* **2006**, *54*, 3686–3692, doi:10.1021/jf052944j.

1. Ueno, H.; Amano, S.; Merecka, B.; Kośmider, J. Difference in the odor concentrations measured by the triangle odor bag method and dynamic olfactometry. *Water Sci. Technol.* **2009**, *59*, 1339–1342, doi:10.2166/wst.2009.112.
2. Cain, W.S.; de Wijk*, R.A.; Jalowayski, A.A.; Pilla Caminha, G.; Schmidt, R. Odor and chemesthesis from brief exposures to TXIB. *Indoor Air* **2005**, *15*, 445–457.
3. Poisson, L.; Schieberle, P. Characterization of the most odor-active compounds in an American Bourbon whisky by application of the aroma extract dilution analysis. *J. Agric. Food Chem.* **2008**, *56*, 5813–5819, doi:10.1021/jf800382m.
4. Nagata, Y. Measurement of odor threshold by triangle odor bag method. *chemestry* **2003**, 118–127.
5. Tamura, H.; Boonbumrung, S.; Yoshizawa, T.; Varanyanond, W. The volatile constituents in the Peel and Pulp of a green thai mango, Khieo Sawoei cultivar (Mangifera indica L.). *Food Sci. Technol. Res* **2001**, *7*, 72–77, doi:10.3136/fstr.7.72.
6. Pino, J.A.; Quijano, C.E. Estudo de compostos voláteis de ameixa (prunus domestica L. cv. horvin) e estimativa da sua contribuição ao aroma. *Cienc. e Tecnol. Aliment.* **2012**, *32*, 76–83, doi:10.1590/S0101-20612012005000006.
7. Peinado, R.A.; Moreno, J.; Bueno, J.E.; Moreno, J.A.; Mauricio, J.C. Comparative study of aromatic compounds in two young white wines subjected to pre-fermentative cryomaceration. *Food Chem.* **2004**, *84*, 585–590, doi:10.1016/S0308-8146(03)00282-6.
8. Komthong, P.; Katoh, T.; Igura, N.; Shimoda, M. Changes in the odours of apple juice during enzymatic browning. *Food Qual. Prefer.* **2006**, *17*, 497–504, doi:10.1016/j.foodqual.2005.06.003.
9. Atanasova, B.; Thomas-Danguin, T.; Langlois, D.; Nicklaus, S.; Chabanet, C.; Etiévant, P. Perception of

wine fruity and woody notes: influence of peri-threshold odorants. *Food Qual. Prefer.* **2005**, *16*, 504–510, doi:10.1016/j.foodqual.2004.10.004.

10. Padrayuttawat, A..; Yoshizawa, T..; Tamura, H..; Tokunaga, T. Optical isomers and odor thresholds of volatile constituents in Citrus sudachi. *Food Sci. Technol. Int. Tokyo* **1997**, *3*, 402–408, doi:10.3136/fsti9596t9798.3.402.
11. Teranishi, R.; Buttery, R.G.; Matsumoto, K.E.; Stern, D.J.; Cunningham, R.T.; Gothilf, S. Recent Developments in Chemical Attractants for Tephritid Fruit Flies. In; 1987; pp. 431–438.
12. Czerny, M.; Christlbauer, M.; Christlbauer, M.; Fischer, A.; Granvogl, M.; Hammer, M.; Hartl, C.; Hernandez, N.M.; Schieberle, P. Re-investigation on odour thresholds of key food aroma compounds and development of an aroma language based on odour qualities of defined aqueous odorant solutions. *Eur. Food Res. Technol.* **2008**, *228*, 265–273, doi:10.1007/s00217-008-0931-x.
13. Kalua, C.M.; Allen, M.S.; Bedgood, D.R.; Bishop, A.G.; Prenzler, P.D.; Robards, K. Olive oil volatile compounds, flavour development and quality: A critical review. *Food Chem.* **2007**, *100*, 273–286, doi:10.1016/j.foodchem.2005.09.059.
14. Takeoka, G.R.; Buttery, R.G.; Ling, L.C.; Wong, R.Y.; Dao, L.T.; Edwards, R.H.; De J Berrios, J. Odor thresholds of various unsaturated branched esters. *LWT - Food Sci. Technol.* **1998**, *31*, 443–448, doi:10.1006/fstl.1998.0382.
15. Siegmund, B.; Pöllinger-Zierler, B. Odor thresholds of microbially induced off-flavor compounds in apple juice. *J. Agric. Food Chem.* **2006**, *54*, 5984–5989, doi:10.1021/jf060602n.
16. van Gemer, L.J. *ODOUR THRESHOLDS*; van Gemert, L.J., Ed.; 2nd ed.; Oliemans Punter & Partners BV: Netherlands, 2011; Vol. 1; ISBN 9789081089401.
17. Hellman, T.M.; Small, F.H. Characterization of the odor properties of 101 petrochemicals using sensory methods. *J. Air Pollut. Control Assoc.* **1974**, *24*, 979–982, doi:10.1080/00022470.1974.10470005.
18. Pickett, J.A.; Birkett, M.A.; Bruce, T.J.A.; Chamberlain, K.; Gordon-Weeks, R.; Matthes, M.C.; Napier, J.A.; Smart, L.E.; Woodcock, C.M. Developments in aspects of ecological phytochemistry: The role of cis-jasmone in inducible defence systems in plants. *Phytochemistry* **2007**, *68*, 2937–2945, doi:10.1016/j.phytochem.2007.09.025.
19. Ferreira, V.; Ardanuy, M.; López, R.; Cacho, J.F. Relationship between Flavor Dilution Values and Odor Unit Values in Hydroalcoholic Solutions: Role of Volatility and a Practical Rule for Its Estimation. *J. Agric. Food Chem.* **1998**, *46*, 4341–4346, doi:10.1021/jf980144l.
20. Von Ranson, C.; Belitz, H.-D. *Originalarbeit Untersuchungen zur Struktur-Aktivitiitsbeziehung bei Geruchsstoffen 3. Mitteilung: Wahrnehmungs-und Erkennungsschwellenwerte sowie Geruchsqualitiiten alicyclischer und aromatischer Aldehyde* *; 1992; Vol. 195;.
21. Takeoka, G.R.; Buttery, R.G.; Turnbaugh, J.G.; Benson, M. Odor thresholds of various branched esters. *LWT - Food Sci. Technol.* **1995**, *28*, 153–156, doi:10.1016/S0023-6438(95)80028-X.
22. Eriksson, C.E.; Lundgren, B.; Vallentin, K. Odor detectability of aldehydes and alcohols originating from lipid oxidation. *Chem. Senses* **1976**, *2*, 3–15, doi:10.1093/chemse/2.1.3.
23. Steinhaus, M.; Sinuco, D.; Polster, J.; Osorio, C.; Schieberle, P. Characterization of the key aroma compounds in pink guawa (Psidium guajawa L.) by means of aroma Re-engineering experiments and Omission Tests. *J. Agric. Food Chem.* **2009**, *57*, 2882–2888, doi:10.1021/jf803728n.
24. Giri, A.; Osako, K.; Okamoto, A.; Ohshima, T. Olfactometric characterization of aroma active compounds in fermented fish paste in comparison with fish sauce, fermented soy paste and sauce products. *Food Res. Int.* **2010**, *43*, 1027–1040, doi:10.1016/j.foodres.2010.01.012.
25. Köster, E.P. Adaptation and cross-adaptation in olfaction: an experimental study with olfactory stimuli at low levels of intensity, Utrecht University: Utrecht, 1971.
26. Ismail, H.M.; Williams, A.A.; Tucknott, O.G. The flavour of plums (Prunus domestica L.). An examination of the aroma components of plum juice from the cultivar victoria. *J. Sci. Food Agric.* **1981**, *32*, 613–619, doi:10.1002/jsfa.2740320614.

27. Buttery, R.G.; Seifert, R.M.; Guadagni, D.G.; Black, D.R.; Ling, L.C. Characterization of some Volatile constituents of carrots. *J. Agric. Food Chem.* **1968**, *16*, 1009–1015, doi:10.1021/jf60160a012.

28. Zeller, A.; Rychlik, M. Character impact odorants of fennel fruits and fennel tea. *J. Agric. Food Chem.* **2006**, *54*, 3686–3692, doi:10.1021/jf052944j.
